# Biomolecular condensates in plant stress and development: recent advances and emerging concepts

**DOI:** 10.1093/jxb/erag288

**Published:** 2026-06-11

**Authors:** Israel Maruri-López, Itzell E Hernández-Sánchez, Malavika Muraleedharan, Monika Chodasiewicz

**Affiliations:** Plant Science Program, Biological and Environmental Science and Engineering Division (BESE), King Abdullah University of Science and Technology (KAUST), Thuwal, Saudi Arabia; Plant Science Program, Biological and Environmental Science and Engineering Division (BESE), King Abdullah University of Science and Technology (KAUST), Thuwal, Saudi Arabia; Plant Science Program, Biological and Environmental Science and Engineering Division (BESE), King Abdullah University of Science and Technology (KAUST), Thuwal, Saudi Arabia; Plant Science Program, Biological and Environmental Science and Engineering Division (BESE), King Abdullah University of Science and Technology (KAUST), Thuwal, Saudi Arabia; University of Illinois, USA

**Keywords:** Abiotic stress, Arabidopsis, biomolecular condensates, LLPS, plant development, RNA-binding proteins, stress granules

## Abstract

Biomolecular condensates have emerged as a central paradigm for understanding how plant cells organize biochemical processes without membrane boundaries, particularly under fluctuating environmental conditions. In plants, many condensates are thought to form through liquid–liquid phase separation, enabling selective concentration of proteins and RNAs into dynamic assemblies. This organization helps buffer stress, protect macromolecules, and reprogram gene expression. Here, we synthesize current knowledge of condensate functions in plant development and stress responses, focusing on *Arabidopsis thaliana*, where mechanistic insights are most advanced. We first outline the biophysical principles underlying condensate formation, emphasizing multivalent protein–protein and protein–RNA interactions, intrinsically disordered regions, and prion-like or low-complexity domains. We then discuss condensates in plant development, highlighting hydration-associated assemblies in seeds, as well as condensation-based regulation of light signaling, auxin pathways, and flowering time. Next, we examine condensates involved in stress adaptation, including stress granules, processing bodies, nuclear assemblies, and chloroplast condensates. Finally, we review the experimental toolkits used to study condensates in plants, including live-cell imaging and fluorescence recovery after photobleaching, *in vitro* reconstitution, proximity labeling, particle enrichment strategies, and RNA-centric profiling approaches, and discuss technical considerations and limitations. We conclude by outlining key open questions regarding condensate regulation, proteostasis, organelle function, and plant resilience under climate change.

## Teaser

Biomolecular condensates, through integration of phase separation, proteostasis, and translational control, emerge as dynamic regulators of plant development, signaling, and stress adaptation to a changing environment.

## Introduction

Biomolecular condensates have emerged as a fundamental principle of cellular organization, reshaping our understanding of how cells compartmentalize biochemical reactions without membranes (Banani *et al*., 2017). Many biomolecular condensates arise through liquid–liquid phase separation (LLPS), a demixing phenomenon that partitions specific molecules into a dense phase co-existing with a surrounding dilute environment (Hofmann *et al*., 2021). By reorganizing molecular interactions in space and time, condensates provide a fast, flexible, and energetically efficient means for plants to perceive environmental signals, protect vulnerable macromolecules, and tune information flow across gene expression, signaling, and metabolism (Peng *et al*., 2025; Wang *et al*., 2025).

In plants, this organizational layer is especially compelling because physiology is constantly negotiated in response to environmental variability. Temperature fluctuations, water limitation, osmotic shifts, oxidative bursts, and nutrient fluctuations impose acute pressures on protein homeostasis and RNA metabolism, while development unfolds across heterogeneous tissues and cellular states (Peng *et al*., 2025; Wang *et al*., 2025). Studies in plants over the last few years have moved the field beyond the question of whether condensates exist, revealing that they participate in processes as diverse as seed maturation and germination, photomorphogenesis, hormone signaling, chromatin-linked regulation, translation control, and organellar homeostasis (Fang *et al*., 2019; Paik and Huq, 2019; Powers *et al*., 2019; Dorone *et al*., 2021). This spatial breadth underscores condensation as a multi-compartment regulatory strategy in plant cells (Field *et al*., 2023).

This review synthesizes current evidence on condensate functions across plant development and stress responses, with a primary emphasis on studies in the model plant *Arabidopsis thaliana*, where mechanistic insights are most advanced. We outline the biophysical principles and molecular features that underpin phase behavior. We then discuss condensate functions in plant development, highlighting hydration-linked assemblies in seeds and condensation-based regulation in light and hormone signaling, flowering control, and nuclear RNA processing. Next, we examine condensates as hubs of abiotic stress response, emphasizing stress granules (SGs), related assemblies, and organellar condensates as emerging regulatory mechanisms such as proteostasis-linked clearance and post-translational control of assembly and disassembly. Finally, we summarize the technological toolbox, including imaging, reconstitution, proximity labeling, and quantitative analysis, together with key caveats that shape interpretation in plant systems.

## Biophysical basis of biomolecular condensates

At the molecular level, condensate formation is governed by molecular concentration and multivalent, non-covalent interactions among proteins and nucleic acids, including electrostatic, hydrophobic, and van der Waals forces, which enable dynamic and reversible assemblies in response to intra- and extracellular fluctuations (Q. Liu *et al*., 2024). These interactions are frequently mediated by intrinsically disordered proteins (IDPs) or proteins containing intrinsically disordered regions (IDRs), Prion-like domains (PrLDs), low-complexity domains (LCDs), oligomerization domains, RNA-binding domains (RBDs), and low-affinity arginine-rich motifs (Maruri-López *et al*., 2021). In addition to proteins, RNA plays a crucial role in condensate nucleation, acting both as a scaffold that increases local interaction valency and as an active component promoting phase separation (Wadsworth *et al*., 2024). Together, protein–protein and protein–RNA interactions provide the physicochemical basis for condensate dynamics and support rapid cellular adaptive responses (Watson *et al*., 2023).

Depending on their molecular composition and cellular context, condensates can adopt a range of physical states, including liquid-like, gel-like (such as semi-fluid gels, glasses, and soft-glasses), or more solid-like assemblies (Boeynaems *et al*., 2018; Hatzianestis *et al*., 2023). Notably, these material properties have been linked to biological function (Jawerth *et al*., 2020). For example, SGs and processing bodies (P-bodies) typically display liquid-like behavior characterized by molecular dynamic rearrangements and fusion events. In contrast, gel-like behavior has been observed in centrosomes, whereas cytoskeletal assemblies such as microtubules are often described as more solid-like assemblies; these physical states are characterized by slower fusion dynamics and reduced or suppressed molecular diffusion (Jawerth *et al*., 2020).

In plants, the majority of biomolecular condensates display liquid-like behavior, with only a limited number of studies reporting gel-like assemblies (Q. Liu *et al*., 2024). Although much of our current understanding of biomolecular condensate formation has emerged from studies in non-plant systems over the past decade, the underlying physical principles governing these processes are rooted in polymer physics and thermodynamics (Jawerth *et al*., 2020; Emenecker *et al*., 2021; Musacchio, 2022). These conserved physicochemical principles operate across eukaryotic cells; however, in plants, they act within a distinct cellular architecture that shapes how condensates acquire specialized biological functions (Emenecker *et al*., 2021).

## Plant cellular architecture shapes condensate behavior

Unlike animal cells, plant cells are characterized by a rigid cell wall, a large central vacuole that restricts cytoplasmic volume, and a dense, dynamic cytoskeletal network (Cosgrove, 2024). These features impose spatial confinement, alter macromolecular crowding, and influence the diffusion landscape, thereby modifying the thresholds, positioning, and material properties of phase-separated assemblies (Banani *et al*., 2017; Emenecker *et al*., 2021). Therefore, plant condensates should not be considered as freely diffusing liquid droplets, but rather as assemblies whose formation and behavior are tightly coupled to cellular architecture.

One example of this coupling is the interaction between condensates and the cytoskeleton. Auxin response factors form cytoplasmic condensates whose dynamics and localization are influenced by actin-dependent transport, linking phase separation to cytoskeletal organization and enabling spatial regulation of signaling outputs (Powers *et al*., 2019; Jing *et al*., 2022). Similarly, the microtubule-associated protein MIDD1 undergoes phase separation along cortical microtubules during xylem differentiation, contributing to microtubule remodeling and secondary cell wall patterning (Higa *et al*., 2024). Together, these studies suggest that cytoskeletal networks can influence the spatial organization and functional behavior of condensates in plant cells.

Membrane interfaces introduce an additional layer of control over condensate behavior. Recent evidence indicates that condensates formed by the ESCRT (endosomal sorting complexes required for transport)-associated protein FREE1 exhibit wetting-like behavior, spreading along endosomal membranes and contributing to multivesicular body biogenesis, membrane invagination, and scission (Y.N. Wang *et al*., 2024). These findings suggest that phase separation can be directly coupled to membrane trafficking processes, expanding the functional repertoire of condensates beyond molecular compartmentalization to include physical remodeling of cellular structures.

Plant cells also contain specialized organelles such as chloroplasts, which impose unique biochemical and physicochemical constraints on condensate assembly. Recent studies have identified stress-induced condensates within chloroplasts, including plastidial SGs and enzyme-associated assemblies involved in photosynthetic regulation, RNA metabolism, and proteostasis under fluctuating environmental conditions (Wu *et al*., 2025; Alquraish *et al*., 2026). These observations further emphasize that condensate behavior is strongly shaped by subcellular compartmentalization.

Collectively, these observations suggest that condensate behavior in plants emerges from an interplay between conserved biophysical principles and the distinctive architectural and physiological features of plant cells. Such interactions are likely to be important for coordinating cellular responses to fluctuating environmental conditions.

## Biomolecular condensates in plant development

Building on their biophysical and molecular principles, the following section focuses on the biological functions of biomolecular condensates in plant systems. Accumulating evidence indicates that condensates participate in diverse cellular processes, ranging from seed maturation and germination, to plant development and flowering, photomorphogenesis, hormone signaling, RNA processing, and responses to biotic and abiotic stresses (Q. Liu *et al*., 2024).

### Seed dormancy and germination

During the plant life cycle, specific developmental stages are characterized by programmed cellular desiccation. Seed development and maturation involve a progressive and tightly controlled loss of up to ∼90% of total cellular water content, a process essential for the acquisition of desiccation tolerance and long-term seed longevity (Leprince *et al*., 2017). As a result, seeds are highly specialized, desiccation-tolerant structures that play a central role in plant lineage perpetuation and successful crop production (Leprince *et al*., 2017). Despite their fundamental biological and agronomic importance, the molecular mechanisms underlying seed maturation and desiccation tolerance have long remained underexplored.

Notably, emerging evidence over the past few years has revealed a prominent role for biomolecular condensates during seed maturation and germination, suggesting that phase-separated assemblies contribute to cellular protection, molecular organization, and the regulation of metabolic reactivation upon imbibition (Field *et al*., 2023). These findings have positioned condensates as a previously underappreciated layer of regulation in seed biology.

Among the molecular components implicated in this process, Late Embryogenesis Abundant (LEA) proteins stand out as a key subject of study. LEA proteins accumulate to exceptionally high levels during late stages of seed maturation, coinciding with minimal cellular water content, and are strongly associated with the acquisition of desiccation tolerance. Although LEA proteins are ubiquitous across the plant kingdom, their occurrence is not restricted to the plant lineage, as they are also reported in bacteria, cyanobacteria, and desiccation-tolerant invertebrates (Ginsawaeng *et al*., 2021a; Field *et al*., 2023).

LEA proteins are compelling candidates for LLPS due to their disordered, low-complexity sequence features and their pronounced structural sensitivity to changes in the solvent environment (Hernández-Sánchez *et al*., 2022; Field *et al*., 2023). In seeds, maturation establishes physicochemical conditions that further favor LLPS-driven condensate formation. Progressive water loss during seed maturation increases ionic strength and macromolecular crowding, thereby promoting the phase separation of certain LEA proteins and potentially influencing the behavior of other biomolecules within the seed cytoplasm. As direct experimental support for this concept, LEA9 was found to form biomolecular condensates with an average diameter of ∼1 µm and liquid-like behavior in mature seeds in a hydration-dependent manner. LEA9 condensates were observed in dissected embryos imbibed for 1 h in water, glycerol, or 2 M NaCl. By contrast, following prolonged rehydration, LEA9 condensates were completely cleared, whereas they persisted under conditions that mimic cellular dehydration (Ginsawaeng *et al*., 2021a, b). Together, these observations indicate that LEA9 condensate formation is a hallmark of the desiccated state of mature seeds, while their dissolution upon rehydration coincides with the onset of germination. This dynamic and hydration-dependent behavior underscores the close coupling between condensate assembly and developmental transitions in seeds. In this context, FLOE1 emerges as an elegant example, highlighting how distinct condensate-forming proteins operate at different stages of the seed hydration–germination continuum (Dorone *et al*., 2021).

FLOE1 is an IDR that functions as a regulator of germination in *A. thaliana* by influencing cellular water content and dynamics, as well as cytoplasmic material properties (Dorone *et al*., 2021; Field *et al*., 2025, Preprint). In contrast to the behavior observed for LEA9, FLOE1 undergoes condensation under hydrated conditions, forming droplet-like puncta of ∼1–2 μm in diameter. Upon water limitation or drying, these condensates dissipate, resulting in a more diffuse cytoplasmic distribution of FLOE1 within seeds (Dorone *et al*., 2021). Through phase transition, FLOE1 regulates water content, dynamics, and cytoplasmic viscosity, linking molecular water control to cellular physical properties.

Together with desiccation-associated LEA condensates, FLOE1 highlights how distinct, hydration-responsive condensate systems operate at different stages of the seed hydration–germination continuum. While direct experimental evidence remains limited, this rapidly developing field offers exciting opportunities to uncover new principles governing cellular organization in seeds.

### Condensate-mediated control of photomorphogenesis and vegetative growth

**Fig. 1.**
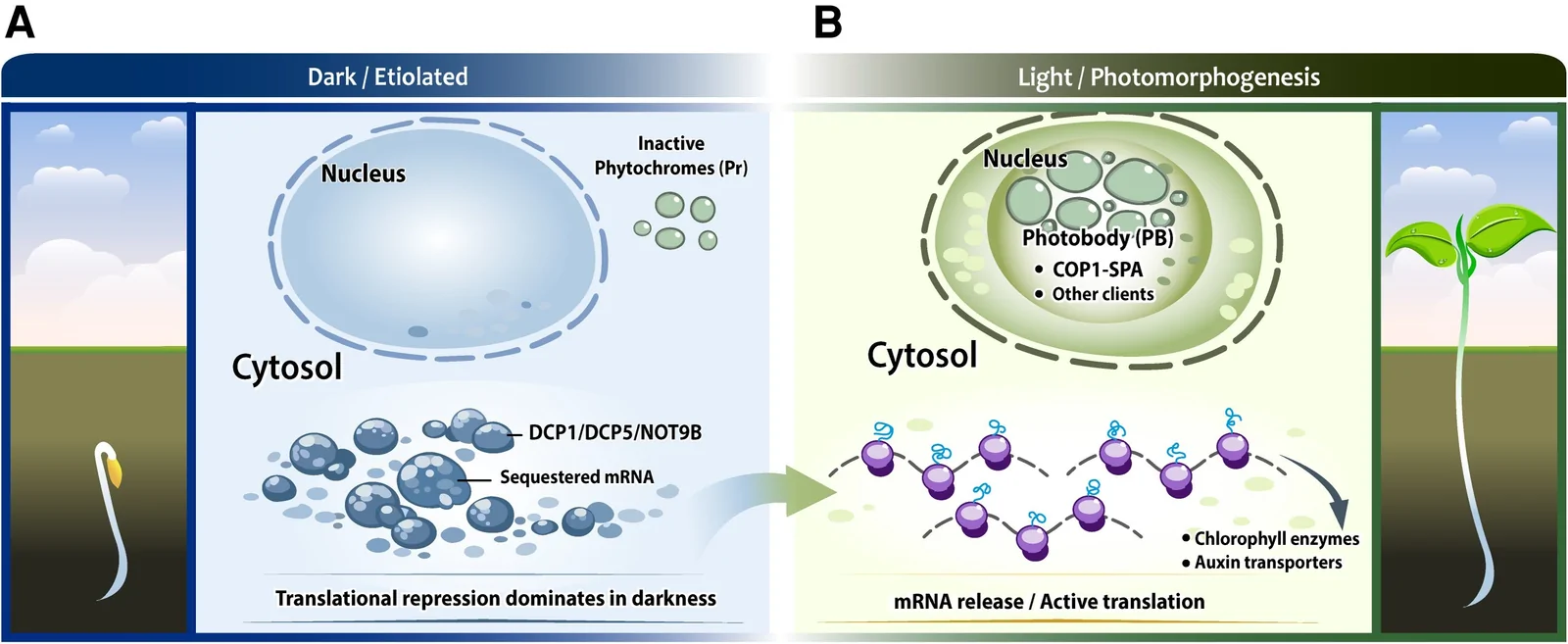
Coordination of nuclear and cytoplasmic condensates during photomorphogenesis. (A) In dark-grown *A. thaliana* seedlings, processing bodies (DCP1/DCP5) accumulate in the cytoplasm, where they sequester translationally repressed mRNAs and limit protein synthesis. (B) Upon exposure to light, phytochromes become activated and translocate to the nucleus, where they assemble into photobodies that act as central hubs for light signaling. In parallel, phytochrome signaling promotes the reduction of cytoplasmic processing bodies (DCP1/DCP5) through COP1- and CCR4–NOT-associated pathways, enabling the release and translation of mRNAs required for photomorphogenic growth, including genes involved in chlorophyll biosynthesis and auxin transport.

Light is a major environmental cue that guides plant development from germination to flowering (F. Wang *et al*., 2024). During early seedling growth in the dark, P-bodies regulate mRNA fate by balancing decay, storage, and translational repression (Chantarachot and Bailey-Serres, 2018). Due to their association with decapping machinery, they have long been viewed primarily as sites of mRNA decay, indicating (Brengues *et al*., 2005; Aizer *et al*., 2014; Hubstenberger *et al*., 2017) that P-bodies also function as transient repositories for translationally repressed mRNAs that can re-enter active translation. In *A. thaliana*, reduced gene expression of a core P-body component, such as DECAPPING PROTEIN 5 (DCP5), reduces P-body abundance and is associated with premature translation of a broad set of mRNAs in dark-grown plants, accompanied by reduced fitness under both dark and light conditions. Upon the transition to light, cotyledon P-bodies diminish in size and number in a phytochrome-dependent manner, coinciding with the release of translationally repressed mRNAs that encode proteins required for photomorphogenic growth and light adaptation. These include enzymes involved in chlorophyll biosynthesis and auxin-related transport processes (Jang *et al*., 2019) (Fig. 1A, B).

While P-bodies act downstream to modulate translational outputs during early seedling development, the initiation of light-dependent developmental programs relies on dedicated photoreceptor systems, including the phytochromes. Arabidopsis encodes five phytochrome photoreceptors (phyA–phyE), with phyB acting as the predominant red/far-red light sensor (Quail, 2002). These photoreceptors are highly conserved across monocots and dicots, and regulate multiple developmental processes, including circadian rhythms, de-etiolation, senescence, and flowering (Paik and Huq, 2019). Upon red/far-red light exposure, phyB shifts from its inactive (Pr) to the biologically active (Pfr) form and moves from the cytoplasm into the nucleus, where it accumulates within subnuclear photobodies (PBs), the primary hubs of phytochrome signaling (Burgie *et al*., 2021) (Fig. 1B). All five Arabidopsis phytochromes localize to PBs, and their light-dependent assembly dynamics are conserved among angiosperms. Notably, strong red light promotes the formation of large PBs, whereas far-red light induces smaller PBs or nucleoplasmic diffusion (Chen *et al*., 2004; Van Buskirk *et al*., 2014).

Beyond their role as signaling hubs, PBs display several characteristics consistent with biomolecular condensates formed via LLPS (Chen *et al*., 2022). PBs exhibit several condensate-like properties, and phyB has been proposed to promote PB assembly through LLPS-related mechanisms in a light- and temperature-dependent manner, with its C-terminal region promoting condensate assembly and the N-terminal extension (NTE) acting as a regulatory module (Chen *et al*., 2022). Although formal *in vitro* reconstitution assays have not yet confirmed LLPS as an intrinsic property of phyB, sequence-based predictions and mutational analyses support the involvement of IDRs within the NTE (Burgie *et al*., 2021). Together, these findings support the view that PBs behave as dynamic condensate-like assemblies whose assembly mechanisms may enable phytochromes to integrate environmental light and temperature cues into transcriptional and developmental outputs.

Consistent with this condensate-like view, proteomic dissection of phyB PBs isolated from *A. thaliana* leaves showed that each PB contains of the order of ∼1500 phyB dimers together with a relatively restricted set of client proteins. These components can be broadly grouped into primary clients, which directly interact with phyB and readily localize to PBs (PIF3/4/7, COP1–SPA1, PCH1/PCHL, ELF3, TZP, phyC, and phyE), and secondary clients, including TOPLESS/TPL-RELATED co-repressors, HSP70 chaperones, and PPK/MLK kinases (Kim *et al*., 2023). This hierarchical organization reinforces the idea that PBs act as scaffold condensates, concentrating phyB and its signaling network to coordinate transcriptional regulation, protein sequestration, and targeted degradation in response to light and temperature, thereby enabling appropriate photomorphogenic responses to the light environment.

Notably, several genetic and molecular observations further link phytochrome signaling to the regulation of cytoplasmic P-body dynamics. Disruption of phytochrome chromophore biosynthesis in the light-insensitive *hy2-106* mutant abolishes the light-induced decrease in P-body size and number, underscoring the requirement for functional phytochrome signaling in this process (Kohchi *et al*., 2001; Jang *et al*., 2019). In parallel, the CONSTITUTIVELY PHOTOMORPHOGENIC 1–SUPPRESSOR OF PHYA-105 (COP1–SPA) complex, a central repressor within phytochrome-mediated pathways (Saijo *et al*., 2003), provides an additional mechanistic link between light perception and translational control. Loss of COP1 function results in constitutive photomorphogenic phenotypes and elevated translation in dark-grown seedlings (Lau and Deng, 2012; Chen *et al*., 2018); consistent with this increased translational status, the P-bodies fail to accumulate in etiolated *cop1* seedlings (Jang *et al*., 2019).

Further supporting this connection, the phyA-interacting protein NOT9B, a component of the CCR4–NOT complex and a negative regulator of light signaling, has been identified as an additional regulator of P-body dynamics (Schwenk *et al*., 2021). NOT9B co-localizes with DCP1-marked P-bodies, and its abundance at the condensate is reduced upon exposure to red and far-red light, simultaneously occurring with the light-induced decrease in P-body markers (Schwenk *et al*., 2021). Moreover, cytoplasmic phyA is required for far-red-dependent reduction of P-body size and number and for proper hypocotyl growth, further reinforcing a direct role for phytochrome signaling in the control of P-body assembly (Schwenk *et al*., 2021). Collectively, phytochromes coordinate light-dependent developmental transitions by orchestrating the dynamic behavior of nuclear PBs and P-bodies, thereby coupling light perception to transcriptional and translational regulation during plant photomorphogenesis (Fig. 1A, B).

### Spatial control of hormone signaling through biomolecular condensates

Auxin signaling relies on precise spatial and temporal control of transcriptional outputs to guide primary and lateral root development (Roychoudhry and Kepinski, 2022). This control is carried out, in part, through biomolecular condensates formed by AUXIN RESPONSE FACTOR (ARF) transcription factors, such as ARF7 and ARF19, which partition signaling capacity by sequestering ARFs away from the nucleus and attenuating transcriptional responses in a context-dependent manner (Powers *et al*., 2019; Jing *et al*., 2022). Condensation of ARF19 impairs its transcriptional activity, effectively acting as a sequestration mechanism that limits auxin signaling. These condensates also display developmental changes in morphology, transitioning from small, spherical assemblies in younger cells to larger, more amorphous structures in differentiated tissues (Powers *et al*., 2019; Emenecker *et al*., 2021).

At the molecular level, ARF19 condensation is driven by multivalent interactions involving its PB1 oligomerization domain and disordered low-complexity regions. Disruption of these interactions abolishes condensate formation and leads to enhanced auxin responses and altered root development. Additionally, ARF19 condensates are regulated by ubiquitin-mediated turnover and the E3 ubiquitin ligase SCF^AFF1^ (Jing *et al*., 2022). They are actively transported along actin filaments by myosin motors, which enhances their growth and stability (Chauhan *et al*., 2025). These findings establish ARF19 condensates as dynamically regulated structures that integrate molecular interactions, cytoskeletal transport, and developmental context to tune auxin-responsive transcription.

A related mechanism has recently been reported in the strigolactone signaling pathway. The signaling repressor SUPPRESSOR OF MORE AXILLARY GROWTH 2-LIKE 7 (SMXL7) forms nuclear condensates that recruit signaling components and transcription factors into repressive assemblies. Within these structures, SMXL7 sequesters transcription factors away from their genomic targets, thereby dampening transcriptional activation of downstream genes. This work suggests that hormone signaling pathways may also use biomolecular condensation to spatially regulate transcription during plant development (Li *et al*., 2025).

### Biomolecular condensates orchestrating the flowering transition

The transition from vegetative growth to flowering represents a major developmental switch that must be precisely timed to ensure reproductive success (Thomson and Wellmer, 2019; Alvarez-Urdiola *et al*., 2025). Notably, recent studies indicate that biomolecular condensation constitutes a broader regulatory principle underlying floral transition and inflorescence architecture, extending beyond individual flowering regulators (Huang *et al*., 2025; Meyer *et al*., 2025).

**Fig. 2.**
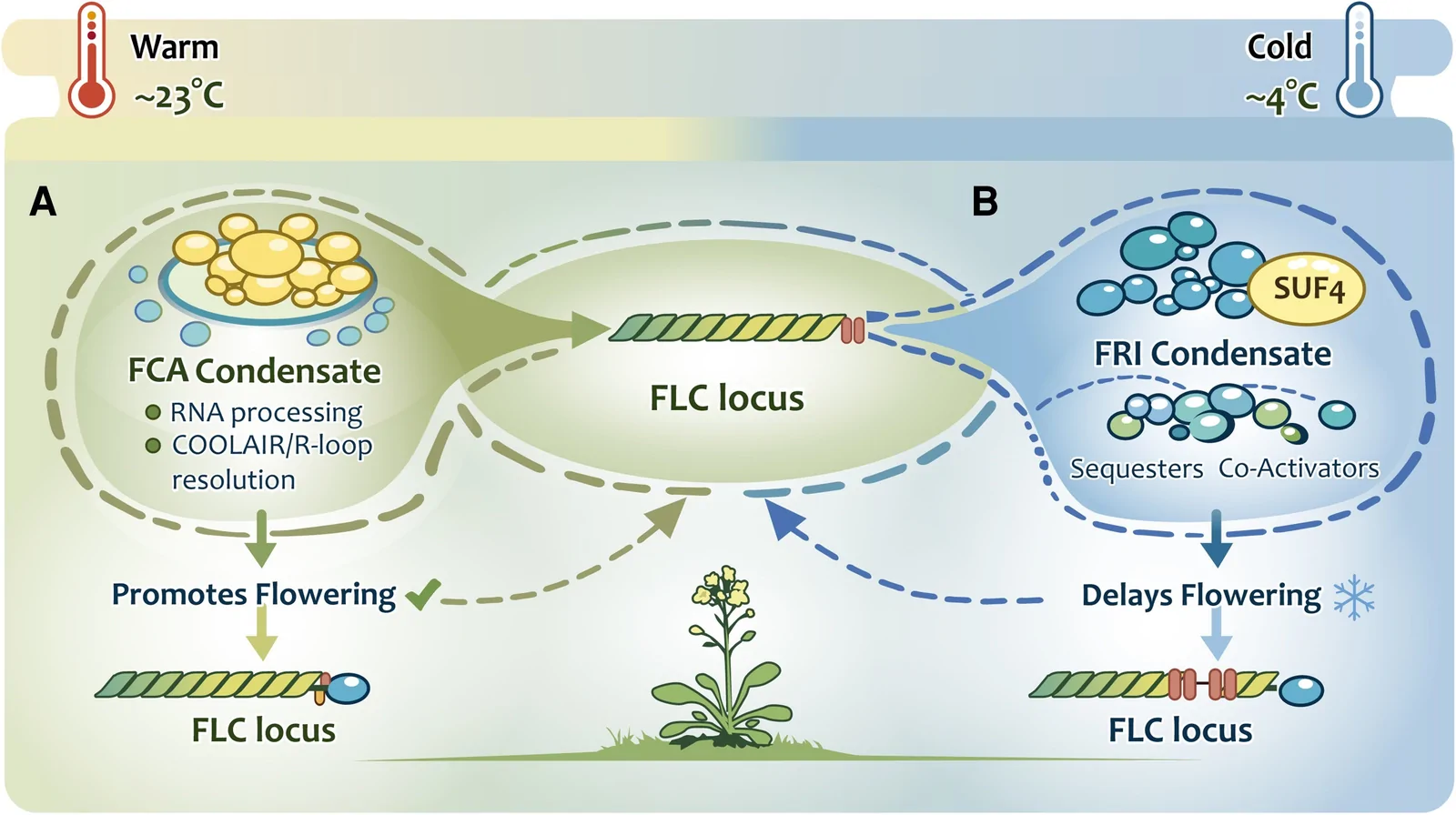
Biomolecular condensates regulate flowering time through opposing control of FLC expression. Biomolecular condensates provide a spatial and temporal framework to regulate the expression of FLOWERING LOCUS C (FLC), a central repressor of flowering in *A. thaliana*. (A) At warm temperatures, the RNA-binding protein FCA forms nuclear condensates associated with 3′-end RNA processing machinery at the FLC locus, promoting COOLAIR proximal polyadenylation and facilitating FLC repression, promoting flowering transition. (B) At cold temperatures, the flowering activator FRIGIDA (FRI) assembles into nuclear condensates that sequester transcriptional activators and RNA processing factors, thereby delaying FLC expression under prolonged cold conditions. The regulator SUF4 also forms nuclear condensates that dynamically reorganize in response to thermal cues, suggesting an additional layer of condensate-mediated control of FLC expression.

In Arabidopsis, flowering time is strongly influenced by the activity of FLOWERING LOCUS C (FLC), a MADS-box transcription factor that functions as a central repressor of floral transition by directly inhibiting the expression of key flowering promoters such as FLOWERING LOCUS T (FT) and SUPPRESSOR OF OVEREXPRESSION OF CONSTANS 1 (SOC1) (Helliwell *et al*., 2006; Searle *et al*., 2006; Deng *et al*., 2011). Modulation of FLC expression constitutes a critical control point for the timing of flowering. Accumulating evidence indicates that FLC is regulated by multiple biomolecular condensates that act at different stages of the transcription cycle and respond to distinct physiological inputs (Fang *et al*., 2019; Zhu *et al*., 2021) (Fig. 2).

One well-known example is provided by FLOWERING CONTROL LOCUS A (FCA), an RNA-binding protein (RBP) that promotes flowering by repressing FLC expression (Fang *et al*., 2019). FCA contains prion-like regions and IDRs that enable it to form liquid-like nuclear condensates together with conserved components of the 3′ RNA processing machinery (Fang *et al*., 2019). FCA-associated 3′-end processing condensates localize near the FLC locus and are proposed to enhance the local dwell time of polyadenylation factors at R-loop-rich regions generated during COOLAIR transcription (Liu *et al*., 2010; Fang *et al*., 2019). Thus, FCA condensates appear to contribute to the establishment of a chromatin environment that reduces FLC transcription (Sun *et al*., 2013; Fang *et al*., 2020; Xu *et al*., 2021) (Fig. 2A). The biological relevance of this mechanism is supported by genetic analyses of mutants that disturb FCA/3′ processing condensate dynamics. These analyses have uncovered contributions from multiple factors, including the coiled-coil protein FLX-LIKE 2 (FLL2), components of the *N*^6^-methyladenosine (m^6^A) RNA modification machinery acting on COOLAIR, and ARGONAUTE1 (AGO1), a protein classically associated with small RNA-mediated regulation (Fang *et al*., 2020). Single-molecule imaging approaches further refined this model by revealing that FCA condensates are built around a tetrameric FCA core that multimerizes into larger, functional assemblies through RNA-dependent interactions and the coiled-coil protein FLL2 (Jang *et al*., 2024, Preprint). These larger condensates display reduced mobility, preferentially recruit 3′-end processing factors, and are required for efficient COOLAIR proximal polyadenylation and FLC repression, providing *in vivo* evidence that condensate size and composition encode regulatory function (Jang *et al*., 2024, Preprint). These findings indicate that FCA condensate function emerges from the coordinated action of RNA processing, RNA modification, and RNA-binding pathways, highlighting an unexpected convergence of post-transcriptional regulatory mechanisms at the FLC locus mediated by biomolecular condensates.

In contrast to FCA, the flowering repressor FRIGIDA (FRI) forms nuclear condensates with different biophysical properties and regulatory roles (Zhu *et al*., 2021) (Fig. 2B). FRI is a positive regulator of FLC expression and plays a central role in delaying flowering until plants have experienced prolonged cold (Zhu *et al*., 2021). FRI condensates assemble in response to low temperature and increase in size and stability during cold exposure, features that correlate with enhanced repression of flowering (Zhu *et al*., 2021). FRI assembles into nuclear condensates through its intrinsically disordered and coiled-coil domains, which recruit FRIGIDA-LIKE1, EARLY FLOWERING 7, and the Cajal body marker U2 SMALL NUCLEAR RIBONUCLEOPROTEIN (Zhu *et al*., 2021). Unlike FCA condensates, FRI assemblies do not appear to associate directly with the transcriptionally active FLC locus. Instead, these condensates restrict, spatially, co-transcriptional activators and RNA processing factors, thereby limiting their availability for FLC activation (Zhu *et al*., 2021) (Fig. 2B). FRI condensates are temperature sensitive, dissolving upon return to warm conditions, highlighting their role as dynamic sensors of environmental cues. Emerging evidence suggests that additional temperature-responsive regulators, such as the IDP SUPPRESSOR OF FRIGIDA 4 (SUF4), may also engage condensation-based mechanisms to modulate FLC transcription. At warm temperatures, SUF4 forms nuclear condensates that co-localize with established flowering regulators, including FRIGIDA and EARLY FLOWERING 7, whereas exposure to cold leads to dispersal of these assemblies into the nucleoplasm, indicating a temperature-dependent shift in condensate organization (Meyer *et al*., 2025) (Fig. 2B).

Together, FCA and FRI illustrate how biomolecular condensates with opposing regulatory roles converge on a shared genetic target to fine-tune flowering time in Arabidopsis (Fig. 2A, B). FCA condensates promote floral transition by facilitating RNA processing and resolving transcription-associated structures at the FLC locus, whereas FRI condensates reinforce repression in response to prolonged cold, contributing to vernalization-dependent control of flowering. The distinct biophysical properties and environmental sensitivities of these condensates indicate that they are engaged at different phases of the transcriptional and developmental cycle, rather than acting concurrently. Through this temporal and mechanistic separation, condensate-mediated regulation provides a flexible framework that integrates intrinsic developmental progression with fluctuating temperature cues to precisely control the timing of the floral transition.

Beyond *A. thaliana*, condensation-based regulation of flowering has also been described in crop species. In tomato, the transcription factor TERMINATING FLOWER (TMF), a key regulator of inflorescence architecture and flowering transition, undergoes reversible redox-regulated phase separation in response to H_2_O_2_ accumulation within the shoot apical meristem (Huang *et al*., 2021). Oxidation-driven disulfide bond formation promotes TMF condensation and sequestration of floral identity loci, thereby delaying meristem maturation and floral transition. Notably, TMF condensates are highly dynamic and reversible, enabling floral timing to respond sensitively to developmental and redox cues. These findings suggest that environmentally responsive condensate mechanisms may contribute to flowering regulation and architectural traits in agriculturally important crops.

### Nuclear speckles and splicing-linked condensates

Nuclear speckles are subnuclear condensates that concentrate factors involved in gene regulation and RNA metabolism, including pre-mRNA processing and mRNA export (Emenecker *et al*., 2021). Although mammalian speckles have been extensively characterized as phase-separated bodies, comparable mechanistic work in plants remains more limited. In mammalian systems, speckles function not merely as storage sites, but as dynamic reservoirs and exchange hubs that regulate the availability of splicing factors, thereby influencing splice-site choice and isoform output.

In *A. thaliana*, emerging evidence suggests that plant speckles may operate under similar regulatory logic. The RS-family splicing factor RSZ22 displays phosphorylation-dependent changes in its mobility and speckle association, implicating kinase activity in the control of SR protein dynamics and subnuclear partitioning (Emenecker *et al*., 2021). Likewise, abscisic acid (ABA)-dependent relocalization of the RBP UBA2 to nuclear speckles indicates that hormonal signaling pathways can actively remodel speckle composition (Emenecker *et al*., 2021). Together, these findings support a model in which nuclear speckles function as signaling-responsive condensates whose assembly state and molecular exchange rates may tune splicing factor availability. Such regulation provides a plausible mechanism by which environmental and hormonal cues could reshape transcript isoform landscapes during growth and stress adaptation.

A clear plant example linking nuclear condensation to developmental gene regulation is the protein Embryo Defective 1579 (EMB1579), which undergoes LLPS *in vitro* and forms highly dynamic nuclear condensates *in vivo* (Zhang *et al*., 2020). EMB1579 is required for normal embryo development, and its loss perturbs global transcription and mRNA splicing during the floral transition. Notably, EMB1579 also influences H3K27me3 deposition at FLC, illustrating how condensate formation can interface with chromatin regulation to coordinate developmental gene expression (Zhang *et al*., 2020). Importantly, the mechanistic reach of nuclear RNA processing condensates in plants extends beyond spliceosomal regulation to small RNA pathways relevant to development. One such entity is the Dicing bodies (D-bodies), nuclear condensates that have been associated with miRNA biogenesis primarily through recruitment of components such as Dicer-like 1 (DCL1) and dsRNA, among others (Fang and Spector, 2007; Xie *et al*., 2021). The phase separation of core Dicing complex protein SERRATE (SE) promotes the organization of D-bodies and subsequent recruitment of miRNA processing factors, enabling efficient miRNA processing to be dependent on this LLPS-mediated assembly (Xie *et al*., 2021). Because miRNAs shape developmental patterning and phase transitions (embryogenesis, meristem identity, and organ morphogenesis) (Axtell and Bartel, 2005; Ma *et al*., 2020), D-body condensation provides a concrete route by which physical state and mesoscale organization can influence developmental gene regulatory circuits (Q. Li *et al*., 2024).

### Biomolecular condensates as central regulators of plant abiotic stress responses

**Fig. 3.**
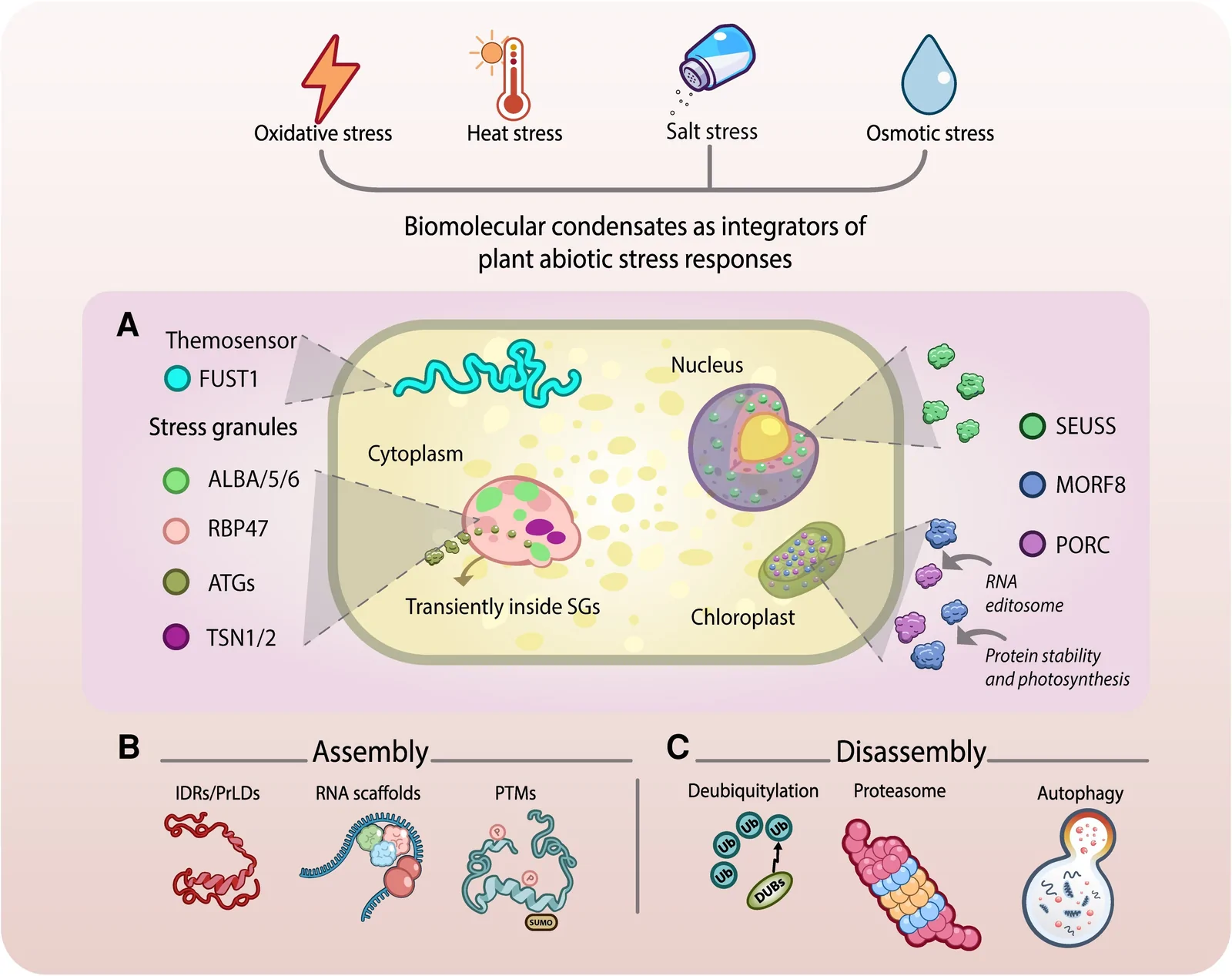
Biomolecular condensates as central regulators of plant abiotic stress responses. Upper panel: diverse abiotic cues, including heat, oxidative, and osmotic stress, trigger the formation of biomolecular condensates across multiple plant cellular compartments. (A) Among these condensates, the best characterized examples are the stress granules, which assemble as dynamic membraneless organelles in the cytoplasm, transiently harboring mRNAs, RNA-binding proteins (RBP47 and ALBA factors), and autophagy-related proteins (ATGs). Condensation extends beyond cytoplasmic constraints. Heat-triggered chloroplastic condensates, including those formed by PORC and MORF8, influence photosynthesis, protein stability, and RNA editing, while osmotic stress triggers SEUSS condensation in the nucleus to support stress-responsive transcriptional reprogramming. (B) Condensate assembly is influenced by sequence-encoded features such as intrinsically disordered or prion-like regions, RNA scaffolds, or post-translational modifications, (C) whereas disassembly involves active clearance and remodeling pathways such as deubiquitylation, proteasome-associated degradation/mechanisms, and autophagy.

SGs and P-bodies remain the most extensively characterized cytoplasmic condensates in plants, serving as core hubs for mRNA triage under stress (Maruri-López *et al*., 2021). SGs assemble in response to translational arrest under diverse stresses (Fig. 3A), whereas P-bodies exist constitutively but change in size and number depending on translational status and environmental cues (Kearly *et al*., 2024). Both structures compartmentalize untranslated mRNAs, RBPs, translation initiation factors, chaperones, and metabolites, providing a flexible means of pausing energy-intensive protein synthesis while protecting stochastic mRNA populations from degradation. Their assembly depends on multivalent protein–RNA interactions (Fig. 3B) that promote rapid and reversible compartmentalization during stress, establishing a dense core enveloped by a more dynamic shell. This architecture enables SGs to rapidly dissolve when stress subsides, ensuring the timely restoration of growth and translation.

In 2019, the first protein composition of heat stress-induced SGs in *A. thaliana* was reported (Kosmacz *et al*., 2019). Subsequent studies expanded the SG proteome, identifying additional proteins that consistently localize to these assemblies under heat stress. For example, Acetylation lowers binding affinity (ALBA)—ALBA4, ALBA5, and ALBA6 (Tong *et al*., 2022)—which was shown to phase-separate into SGs and P-bodies under heat stress, directly brings selected mRNAs (including heat shock factor mRNAs) into SGs and P-bodies. The role of this mechanism is to protect the heat shock factor mRNA from degradation under heat stress conditions (Fig. 3A). Another example is Tudor staphylococcal nuclease 1 and 2 (TSN1/2) that localizes into SG and P-bodies under heat and salinity stress (Gutierrez-Beltran *et al*., 2021). The number identified as components of SGs under salinity stress was much smaller in comparison with heat stress-induced SGs. Expanding beyond *A. thaliana*, a recent study in rice further highlights the translational relevance of SG-mediated regulation in crops. H.J. Wang *et al*. (2024) identified the dsRNA-binding protein DROUGHT RESISTANCE GENE 9 (DRG9), which undergoes phase separation into SGs under drought stress conditions. DRG9 selectively recruits and stabilizes stress-responsive transcripts, including OsNCED4, a key ABA biosynthesis gene, thereby protecting these mRNAs from degradation during drought stress. Notably, natural allelic variation in DRG9 enhances drought resistance in rice, and introgression of the drought-resistant allele into elite cultivars significantly improves stress tolerance. These findings provide a compelling example of how condensate-mediated RNA regulation can directly contribute to agronomically relevant traits.

A groundbreaking discovery further expanded the known functions of SGs by directly linking them to the regulation of autophagy and proteostasis. X.B. Li *et al*. (2024) demonstrated that during heat stress, key autophagy proteins, including ATG1a, ATG13a, ATG6, VPS34, and ATG5, translocate into SGs, which act as temporary repositories that prevent premature activation of autophagy by providing a spatially coordinated site for autophagy regulators to accumulate (Fig. 3A). As heat stress diminishes, these ATG proteins are rapidly released back into the cytoplasm, enabling efficient initiation of autophagic clearance of ubiquitinated protein aggregates. An Arabidopsis mutant impaired in SG formation displayed pronounced delays in autophagy activation and compromised protein quality control, revealing that SGs serve as a regulatory checkpoint that tightly couples translational repression to proteostasis recovery. This finding underscores an emerging principle: condensates can integrate multiple stress response pathways, ensuring synchronized cellular rehabilitation after stress release.

Heat stress has also revealed a remarkable diversity of condensate-mediated sensing mechanisms in plants. A particularly compelling example is the discovery of the protein FUST1 as a direct molecular thermosensor (Geng *et al*., 2025) (Fig. 3A). The authors showed that FUST1 undergoes rapid condensation within minutes of temperature elevation, forming cytoplasmic foci just before any SG markers appear. Molecular dynamics simulations revealed that its prion-like domain acts as a thermo-switch, transitioning from a locked to an open conformation as temperature rises, thereby promoting multivalent interactions that drive LLPS. Loss of FUST1 protein severely impaired SG dynamics and reduced both basal and acquired heat tolerance, demonstrating that thermosensing through conformationally driven condensation constitutes a primary mechanism through which plant cells initiate cytoplasmic reorganization during heat exposure. This discovery represents one of the clearest examples of a plant protein whose sequence-encoded biophysical properties directly translate temperature changes into cellular action.

Interestingly, heat-induced condensation also occurs within chloroplasts. The first report in 2020 identified chloroplast-localized foci that are formed in response to high temperature stress and resemble properties of cytosolic SGs (Fig. 3A). Therefore, they were called plastidial SGs (Chodasiewicz *et al*., 2020). Recently, it has been shown that Protochlorophyllide Oxidoreductase C (PORC), a chlorophyll biosynthesis enzyme, forms condensates in response to high temperature treatment, and those condensates recruit proteins involved in protein stability and photosynthesis, serving as a protective mechanism under stress (Alquraish *et al*., 2026) (Fig. 3A). Overexpression of PORC led to improved tolerance, particularly under prolonged heat stress conditions. Notably, more examples of plastidial condensation have been discovered, revealing that chloroplast-associated condensates can exhibit distinct material properties. Wu *et al*. (2025) uncovered that MORF8, a key component of the chloroplast RNA editosome, forms solid-like condensates specifically under heat stress. Unlike liquid-like cytoplasmic SGs, MORF8 condensates display little fluorescence recovery after photobleaching, resist dissolution, and persist long after heat removal, suggesting a transition toward a more rigid, gel-like state. MORF8 condensation sequesters multiple RNA-editing factors and significantly reduces editing efficiency at ndhD sites, thereby impairing NADH dehydrogenase-like (NDH) complex function and weakening cyclic electron flow around PSI (Fig. 3A). This chain of events ultimately diminishes photosynthetic efficiency, providing a molecular explanation for the long-observed decline in chloroplast performance under elevated temperatures. The discovery of solidifying organellar condensates introduces a novel concept in which LLPS not only protects or reorganizes macromolecules but can also induce stress-induced functional silencing within organelles.

Beyond cytoplasmic and chloroplastic contexts, stress-induced condensates were also identified in the nucleus, for example in response to osmotic stress. SEUSS (SEU), a transcriptional regulator, forms nuclear condensates within minutes of hyperosmotic treatment (B.Y. Wang *et al*., 2022) (Fig. 3A). Wang *et al*. demonstrated that SEU condensation is driven by IDR1, which undergoes crowding-induced compaction under conditions mimicking osmotic shrinkage, thereby promoting phase separation. These condensates are essential for activating stress-responsive transcriptional programs and dissolve rapidly upon osmotic recovery. The specificity of SEU condensation, occurring under osmotic stress but not heat, highlights the exquisite stimulus sensitivity encoded in condensate-prone proteins, allowing plants to discriminate among different environmental cues through distinct biophysical signatures.

While condensate formation is critical for stress adaptation, timely disassembly is equally important for restoring cellular balance. Pang *et al*. (2025) identified the AP-3β subunit as a key factor in SG disassembly during heat recovery, acting through its interaction with TSN1/2 to recruit the 19S regulatory particle of the proteasome to SGs (Fig. 3C). Notably, the proteasome promotes disassembly not through proteolysis but through associated deubiquitylation, targeting the K63-linked polyubiquitin chains enriched in heat-induced SGs. Mutants lacking AP-3β exhibit persistent SGs, delayed translation reinitiation, and impaired post-stress growth. This reveals a mechanistically unique pathway in which endomembrane trafficking components interface with the ubiquitin–proteasome system to modify condensate dynamics, adding to the growing appreciation that condensates require active, regulated clearance. Control of condensate assembly and dissolution is further refined through post-translational modifications (PTMs). Legoux *et al*. (2025) highlight the roles of phosphorylation, ubiquitination, acetylation, and SUMOylation in modulating condensate material properties, interaction networks, and assembly thresholds (Fig. 3C). Phosphorylation can induce structural rearrangements that weaken or strengthen LLPS-driving interactions. Ubiquitination, particularly K63-linked chains, serves as a selective signal for recruitment into or removal from SGs; and SUMOylation may tune RNA binding affinity or solubility of condensate-associated proteins (Fig. 3C). These regulatory mechanisms endow condensates with remarkable responsiveness, enabling cells to fine-tune their internal organization with temporal precision through reversible biochemical modifications.

Collectively, the studies reviewed here converge on a unifying picture of plant condensates as highly adaptable molecular machines that translate environmental cues into coordinated biochemical and structural responses. They function as thermal sensors, osmotic sensors, organizers of translation and mRNA metabolism, regulators of autophagy, modulators of organellar gene expression, and even drivers of membrane remodeling. Their rapid assembly and dissolution rely on both non-covalent interactions and intricate layers of PTM-based regulation, while their disassembly can involve proteasome-associated deubiquitylation or other active mechanisms. As research into plant condensates expands, new classes of stress-responsive assemblies are likely to be uncovered, and their integration into broader cellular networks will continue to illuminate innovative mechanisms by which plants survive an increasingly unpredictable environment.

## Technological approaches to studying plant biomolecular condensates

The rapid expansion of biomolecular condensate research has been driven not only by conceptual advances but also by the development of a diverse set of experimental tools that enable visualization, reconstitution, and quantitative characterization of phase-separated entities. In plants, the application of these approaches presents unique challenges, including cellular complexity, tissue heterogeneity, and more. Nevertheless, the ever-expanding suite of imaging, biochemical, and biophysical methods has made it increasingly feasible to study the condensate formation, dynamics, and function *in vivo* and *in vitro*. Importantly, while many of these tools were primarily developed in animal or yeast systems, their adaptation to plant biology has begun to reveal conservation as well as plant-specific principles of condensate organization. Here, we highlight some essential tools successfully used in plant condensate biology, while key methodological considerations and recommended practices for validating plant biomolecular condensates are summarized in Box 1.

Box 1. **Toolbox: operational considerations for validating plant biomolecular condensates.**

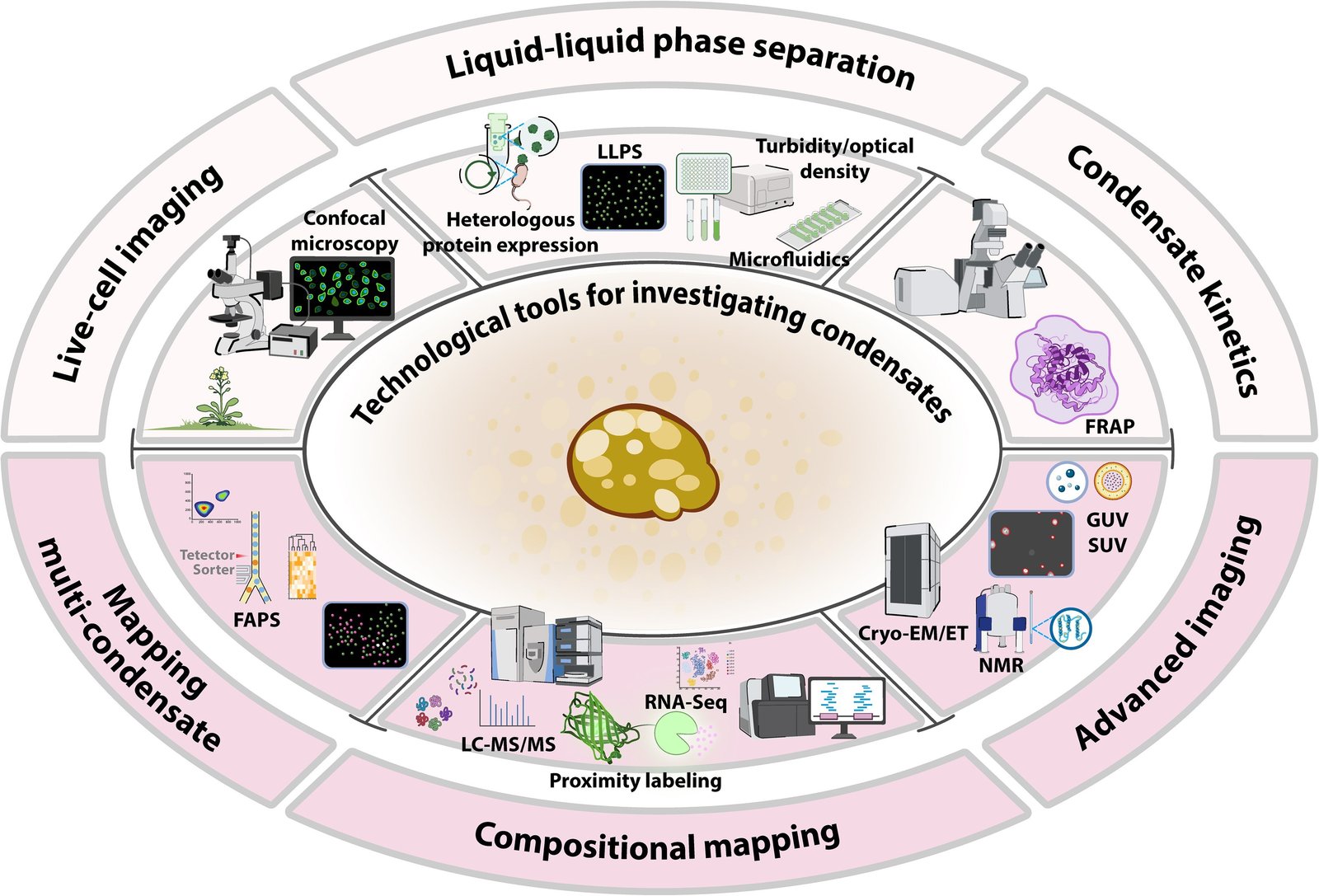
A central methodological problem in plant condensate research is that puncta formation is visually obvious but mechanistically ambiguous. To establish LLPS or related phase behaviors, best practices rely on integrating multiple orthogonal lines of evidence, including reversibility, concentration dependence, molecular mobility, compositional profiling, and other complementary assays. The emergence of foci alone may not be sufficient to demonstrate phase separation or distinguish reversible demixing from aggregation, material reorganization from simple accumulation, or regulated compositional remodeling from generic stress-induced crowding. Because condensate behavior is concentration and context dependent, small changes in expression level or cellular state can shift apparent thresholds and generate puncta without altering the underlying physical regime (Hatzianestis *et al*., 2023; Alberti *et al*., 2025).

Therefore, puncta visualization should be complemented with approaches that provide additional information about condensate internal dynamics and material properties. Among these, FRAP remains one of the most widely used methods to assess molecular mobility within condensates. Recovery curves integrate exchange with the dilute phase, internal rearrangement, binding equilibria, and bleaching geometry. However, fast recovery does not necessarily indicate liquidity, and slow recovery does not by itself demonstrate solidification. Interpretation requires quantitative comparison across perturbations that shift concentration or interaction strength. For this reason, FRAP should be treated as one parameter within a broader material state assessment that includes fusion dynamics, partitioning behavior, and, where possible, measurements sensitive to viscoelastic transitions (Ray and Buell, 2024; Ji *et al*., 2025).

While imaging-based approaches provide important information about condensate dynamics *in vivo*, they often cannot directly resolve the molecular interactions driving assembly. In this context, *in vitro* reconstitution provides a critical test of sufficiency, but its interpretive strength depends on experimental design. Recombinant systems can define how variables such as ionic strength, macromolecular crowding, RNA, or ligands modulate demixing behavior. However, non-physiological protein concentrations, affinity tags, and buffer composition can shift phase boundaries and generate regimes not encountered *in vivo*. Physiological relevance depends on demonstrating that the *in vitro* system operates within concentration and stoichiometric regimes that are quantitatively compatible with the cellular environment. Best practice includes systematic phase diagram sampling, quantitative morphometric analysis rather than representative micrographs, and explicit controls for labeling, purification artifacts, and mechanical stress introduced during sample processing (Ray and Buell, 2024; Alberti *et al*., 2025).

In addition to reconstitution assays, chemical perturbation approaches can provide useful insights into the molecular interactions contributing to condensate stability and material behavior. In this scenario, 1,6-hexanediol is used to disrupt weak hydrophobic interactions. Sensitivity to hexanediol does not establish phase separation, and resistance does not exclude it. Hexanediol can perturb membranes, alter enzyme activity, and induce cellular stress, complicating interpretation. Perturbation responses should therefore be analyzed alongside concentration dependence, reversibility, and independent material-state measurements, rather than treated as a diagnostic criterion for LLPS (Alberti *et al*., 2025; Zhu *et al*., 2025).

Advanced imaging and mechanics are defining the next phase of the field. Super-resolution and correlative workflows are increasingly used to test whether diffraction-limited uniform punctae contain substructures (cores, shells, multiphase organization), while cryo-electron tomography and related ultrastructural approaches aim to describe condensate architecture in near-native states rather than fixed proxies. In parallel, viscoelastic readouts (rheological measurements or fusion relaxation analysis) are becoming the preferred way to document liquid-to-gel/solid transitions when morphology alone is ambiguous (Hatzianestis *et al*., 2023; Alberti *et al*., 2025).

Finally, quantification is no longer optional. Measuring condensate volume, partitioning, and internal concentration is necessary because condensate behaviors are often threshold driven: progressive up-concentration can precede demixing or hardening events that are not captured by puncta counts. The field is converging on standardized segmentation-based pipelines and explicit reporting of analysis parameters to improve reproducibility and comparability across labs and systems (Smokers and Spruijt, 2025).

### Live-cell imaging microscopy

Live-cell fluorescence microscopy remains a cornerstone of condensate research, providing direct visualization of subcellular compartmentalization in real time. In plants, confocal imaging of fluorescently tagged components in stable lines has enabled routine trafficking or visualization of cytosolic and nuclear punctate structures such as SGs, P-bodies, nuclear speckles, and other RNA- or protein-rich assemblies across tissues and developmental stages. Transgenic lines expressing fluorescently tagged condensate components under endogenous or inducible promoters allow spatial and temporal analysis of condensate behavior across tissues and developmental stages. A key advantage of live-cell imaging is the ability to monitor condensate dynamics in response to environmental or developmental cues, such as heat stress, nutrient deprivation, hormonal signaling, and more. For example, live-cell imaging of FUST1 in Arabidopsis showed that heat triggers autonomous FUST1 condensation before the recruitment of several established SG markers, showcasing how real-time microscopy continues to aid in characterizing beyond the descriptive puncta imaging to resolve the temporal hierarchy and mechanistic basis of condensate assembly under stress (Geng *et al*., 2025). Time-lapse confocal imaging holistically outputs high-standard descriptive reports covering stress titration (e.g. graded heat, oxidative, and osmotic), temporal kinetics (assembly and disassembly), spatial localization (nuclear, cytosolic, and organellar), and context dependence (developmental stage, tissue type, and recovery).

FRAP (fluorescence recovery after photobleaching) remains the workhorse for verifying the liquid character and molecular mobility of condensates (Hatzianestis *et al*., 2023). A defined region is bleached, and recovery kinetics are tracked to infer mobile fractions and exchange time scales, thereby distinguishing among liquid-like, gel-like, and more solid-like states (Brangwynne *et al*., 2009; Shin and Brangwynne, 2017). However, when used alone, this approach cannot fully resolve the fundamental biophysical mechanisms or material properties of condensates and therefore should be complemented with techniques capable of disentangling internal molecular mixing from exchange with the surrounding dilute phase (Ray and Buell, 2024; Ji *et al*., 2025).

As data synthesis moves from puncta formation to its quantification, fluorescence correlation spectroscopy (FCS) and related fluctuation approaches are increasingly valuable for estimating concentration, diffusion, and molecular interaction states within and outside condensates (Z. Wang *et al*., 2022). A complementary readout for FCS is to pair it with Förster resonance energy transfer (FRET)-derived proximity readouts, where these techniques monitor recruitment, releases, and nanoscale proximity of components within condensates. However, these techniques in plant use are not widely established and may be perturbed by plant-specific imaging constraints, such as autofluorescence artifacts. Therefore, it is key to design experiments that complement the structural complexity and material sensitivity of the specimen at hand.

### *In vitro* phase separation assays

Plant condensate frameworks explicitly place *in vitro* reconstitution at the center of mechanistic dissection, mostly to probe whether a candidate protein (often enriched in IDRs/PrLDs) is sufficient to form a condensate without the perturbation/influence of plant internal complexities. Proteins are expressed (e.g. *Escherichia coli* or yeast), purified, and assayed across controlled variables such as crowding agents [polyethylene glycol (PEG)], salts (NaCl/KCl), nucleic acids (RNA/DNA), metal ions, and small molecules, enabling the mapping of interaction logic that drives condensation (Emenecker *et al*., 2021). For example, plant developmental regulators such as the seed protein FLOE1 undergo *in vitro* phase separation in a sequence-dependent manner, enabling a reconstitution to physiology link (Dorone *et al*., 2021). *In vitro* reconstitution, therefore, allows controlled manipulation of physicochemical parameters and direct testing of domain or motif contributions (X. Wang *et al*., 2024). Common readouts for *in vitro* phase separation assays include microscopical droplet imaging and morphology tracking complemented with measuring turbidity (optical density) and mobility kinetics (FRAP). A key bridge between qualitative imaging and quantitative output is image-based segmentation and standardized measurement of droplet features (count, size distributions, intensity/partitioning, circularity, and temporal evolution). The phase metrics workflow is one example of an imaging-based quantification pipeline used to extract the above-mentioned features (Ray and Buell, 2024).

Microfluidic approaches are becoming central for systematically exploring phase diagrams (rapid mixing, controlled gradients of salt/crowder/ligand, and high-throughput sampling of conditions) and for interrogating mechanical effects that are hard to control in bulk. These approaches offer control over mixing, timing, and composition, allowing condensate formation to be triggered in a precise and reproducible manner (Ray and Buell, 2024). Droplet microfluidics can generate thousands of minuscule reaction compartments to efficiently sample space and approximate phase boundaries.

In the realm of phase separation assays, hexanediol remains a widely used droplet perturbator as it disrupts weak hydrophobic interactions and often reduces turbidity in LLPS systems. However, in recent times, multiple reviews emphasize strong caveats to using hexanediol due to its insensitivity to many interaction types (electrostatics, cation–π, and π–π), off-target effects on enzymes, membrane permeability changes, and non-specific cellular disruption. Consequently, hexanediol should be positioned as a supporting perturbation rather than a decisive test (Zhu *et al*., 2025).

## Mapping condensate composition: proximity, enrichment, and RNA-centric strategies

Defining condensate composition is a prerequisite for understanding its biological function. In plant systems, this has required methodological approaches capable of capturing dynamic and stress-sensitive molecular assemblies with molecular specificity. Numerous complementary strategies have emerged for this purpose. In this section, we turn to a more detailed examination of proximity-based labeling to record the local molecular environment of condensate scaffolds directly in living cells. We further examine a second method that depends on the physical enrichment of condensate-positive particles before downstream proteomic or RNA analysis. We finally examine a third approach focused specifically on defining RNA cargo selectivity, an essential determinant of condensate function. Together, these approaches provide an integrated framework for mapping condensate composition with increasing spatial, molecular, and functional resolution.

### Proximity-based mapping of condensate neighborhoods

Defining condensate composition is central for moving from marker co-localization to unveiling the mechanism. However, condensate-associated interactions are frequently weak, stress dependent, and may be lost during lysis. Consequently, plant condensate workflows commonly integrate enrichment-based proteomics with *in situ* proximity labeling strategies that preserve local molecular neighborhoods during the relevant physiological state. Proximity labeling approaches, including biotin ligase-based methods such as TurboID, are particularly suited for condensates because they can capture transient and low-affinity associations without requiring stable biochemical recovery of intact assemblies (Mair *et al*., 2019). In *A. thaliana*, TurboID proximity labeling was applied to characterize the composition of heat-induced FUST1 condensates/SGs, explicitly leveraging the short-lived nature of SG interactions under stress (Geng *et al*., 2025). Similarly, the combined mTurboID proximity labeling with multi-omics approaches has helped define the interactome of the RBP47b SG marker. Thus, the TurboID assay identified a stress-specific enrichment of 40S ribosomal subunits within RBP47b SGs, implicating these condensates in translational control (Hernández-Sánchez *et al*., 2025, Preprint). However, it is noteworthy that this approach reports proximity rather than direct binding; this limitation is rescued when paired with appropriate controls (spatial controls, inactive enzyme controls, and non-stress controls) and follow-up validation of candidates by microscopy and complementary assays.

### Particle enrichment and physical isolation of condensates

When condensates are fluorescently labeled, fluorescence-activated particle sorting (FAPS) provides a conceptually clean enrichment step; cells can be lysed under conditions that preserve particles, and fluorescent condensate-positive events are physically sorted away from granule-free material before downstream proteomics or RNA profiling. The foundational demonstration of this logic came from purification of endogenous P-bodies by FAPS, enabling systematic definition of their protein and RNA content while reducing background from bulk lysate (Hubstenberger *et al*., 2017). Subsequent methodological advances have formalized FAPS pipelines for condensate purification, refined gating strategies, addressed sample handling constraints, and ensured compatibility with MS and RNA-seq workflows (Godebert *et al*., 2026).

Building on this framework, FAPS has been successfully adapted to plant systems. In *A. thaliana*, lysates from salicylic acid-treated seedlings expressing ECT1–green fluorescent protein (GFP) condensates were purified by FAPS to define the compositional signatures of specific stress-responsive puncta (Lee *et al*., 2024). In plant contexts, where tissue heterogeneity and low condensate abundance present recurring challenges, FAPS could be viewed as an omics-enabling enrichment module rather than a stand-alone assay. By increasing specificity before LC-MS/MS and RNA profiling, and when paired with parallel pre- and post-sort microscopy validation, it allows confident assignment of molecular content to intact condensate-like particles.

### RNA-centric profiling

RNA selectivity is increasingly recognized as a central principle underlying condensate biology and function. With condensates harboring diverse species of RNA, defining their RNA composition is essential for understanding condensate assembly, specificity, and biological roles (Van Treeck *et al*., 2018). Initial approaches have relied on sequencing RNAs recovered from enriched condensate fractions, complemented by orthogonal imaging-based validation methods. However, these methods can under-represent transiently associated or weakly retained transcripts. At the broadest level, enrichment or proximity-based approaches aim to define RNAs present within the condensates or their immediate environment. For instance, Turbo RNA immunoprecipitation (T-RIP), a TurboID-based proximity capture strategy, has enabled the identification of RNAs associated with P-bodies in *A. thaliana*. This approach revealed that P-body-associated transcripts are highly dynamic and are enriched for genes involved in developmental regulation, secondary metabolism, and immune responses, among other processes (C. Liu *et al*., 2024). It is important to note that proximity-labeling and RIP-based approaches generally rely on chemical fixation or indirect recovery of ribonucleoprotein (RNP) complexes rather than directly identifying RNA–protein interaction sites (Lewinski *et al*., 2024). In the dense environment of biomolecular condensates, distinguishing direct RBP–RNA targets becomes particularly important for accurate interpretation. To address these limitations, UV cross-linking-based CLIP (cross-linking and immunoprecipitation) methods have been developed. In plants, the establishment of high-throughput sequencing of RNA isolated by cross-linking immunoprecipitation (HITS-CLIP) provided an early framework for recovering direct RBP-bound RNAs through UV-induced crosslinking followed by immunoprecipitation. This approach enabled the identification of RNAs associated with the hnRNP A/B-like protein HLP1, linking its RNA targets to the regulation of flowering time in *A. thaliana* (Zhang *et al*., 2015). HITS-CLIP resolves protein–RNA interactions at the level of short RNA regions rather than precise nucleotide positions, which can limit mechanistic interpretation and highlight the need for higher-resolution CLIP variants. Recent efforts in condensate–RNA profiling have therefore focused on establishing approaches with improved resolution *in planta*. For example, individual-nucleotide resolution CLIP (iCLIP), and its improved variant iCLIP2, have recently been implemented in plants as a framework for transcriptome-wide mapping of RBP–RNA interactions at near single-nucleotide resolution (Lewinski *et al*., 2024).

In parallel, editing-based approaches have expanded the toolkit for condensate RNA mapping. Methods such as Hyperactivated Target of RNA-binding proteins identified by editing (HyperTRIBE) are used to profile RNAs associated with an RBP by fusing it to a hyperactive ADAR catalytic domain, such that nearby RNAs acquire diagnostic A-to-G changes detectable by RNA sequencing (Zhou *et al*., 2021). In plants, comparisons of HyperTRIBE and iCLIP datasets showed that HyperTRIBE can recover weakly expressed targets that are often under-represented in CLIP-based assays (Lewinski *et al*., 2024). Extending this principle, HyperADARcd fused to AtUBP1c was used to profile AtUBP1c-associated RNAs during immune activation, revealing a strong increase in edited target RNAs upon activation of plant immune responses (Zhou *et al*., 2021). Taken together, these approaches highlight that no single method can fully capture RNA biology within biomolecular condensates. Instead, the most informative mechanistic insights will probably arise from integrative strategies that combine systematic cataloging of condensate-associated RNAs with functional analyses linking changes in RNA composition to downstream outcomes, such as altered translation, RNA processing, or developmental phenotypes, under carefully controlled stress or developmental conditions.

## Conclusion and future directions

In this review, we have synthesized emerging evidence that biomolecular condensates constitute a layer of organization in plant cells, which operates across development and stress adaptation. From hydration-linked assemblies in seeds to light- and temperature-responsive nuclear PBs, to cytoplasmic SGs and organellar condensates, plant condensates appear frequently as dynamic control points that tune gene expression, translation, proteostasis, and signaling. Collectively, these studies move the field beyond the question of whether plant condensates exist and toward the more consequential questions of when condensation is causal, what molecular features encode stimulus sensitivity, and how material state and composition specify biological output.

In *A. thaliana*, condensate formation is often associated with a phenotype, but clear evidence that condensation itself drives that outcome remains limited. Addressing this gap depends on experimental designs that uncouple phase behavior from other biochemical activities of the same protein. Genetic variants that selectively alter interaction interfaces, multivalency, or regulatory residues can shift condensation propensity while preserving overall protein levels and basal function. Interpreting these perturbations requires quantitative analysis of concentration dependence, reversibility, and material transitions, so that sequence features that promote condensation can be linked directly to physiological outputs *in vivo.*

The field also needs sharper models for how plants encode stimulus specificity into condensation. Several examples now suggest that condensates can function as physical sensors, such as temperature, osmotic shrinkage, hydration state, or proteostasis imbalance, yet the general rules remain unclear. Future studies should map how post-translational modifications, RNA availability, chaperone buffering, and cytoskeletal transport reshape phase boundaries and kinetics in living plant cells. Equally important will be to define how condensates integrate with core cellular processes: how assembly competes with translation and degradation, how disassembly is actively driven during recovery, and how organelles and condensates coordinate under combined stresses that more closely resemble natural environments. Particularly promising directions include determining how liquid-like and solid-like material states influence membrane-associated condensates, organellar function, and stress recovery dynamics, as well as identifying the molecular features that control these transitions *in vivo*.

Technological innovation will continue to set the pace of discovery, but plant-specific constraints remain non-trivial. Cell walls, autofluorescence, tissue heterogeneity, and stress can complicate quantitative imaging and material-state inference. Future advances will be likely to come from integrating orthogonal toolkits, such as super-resolution and correlative ultrastructure to resolve internal organization; mechanics-sensitive readouts to distinguish liquid-like exchange; microfluidics and high-throughput reconstitution to map phase diagrams; and proximity labeling, particle sorting, and RNA-centric approaches to capture labile composition.

In summary, plant condensate biology is transitioning from a descriptive phase to a mechanistic discipline that links mesoscale organization to development, stress resilience, and organellar function. As discovery expands and causal frameworks mature, condensate principles are poised to inform new strategies for engineering stress tolerance and productivity by rewiring stimulus-responsive assembly, stabilizing recovery dynamics, or tuning translation and proteostasis through programmable compartmentalization in a changing climate. In the longer term, rational engineering of IDRs and multivalent interaction modules may provide new opportunities to modulate condensate behavior and improve crop resilience under fluctuating environmental conditions.
